# Molecular characterisation of influenza B virus from the 2017/18 season in primary models of the human lung reveals improved adaptation to the lower respiratory tract

**DOI:** 10.1080/22221751.2024.2402868

**Published:** 2024-09-09

**Authors:** Duygu Merve Çalışkan, Sriram Kumar, Saskia Hinse, Klaus Schughart, Rainer Wiewrodt, Stefan Fischer, Vera Krueger, Karsten Wiebe, Peter Barth, Alexander Mellmann, Stephan Ludwig, Linda Brunotte

**Affiliations:** a Institute of Virology, University of Münster, Münster, Germany; b EvoPAD Research Training Group 2220, University of Münster, Münster, Germany; c Department of Microbiology, Immunology and Biochemistry, University of Tennessee Health Science Center, Memphis, TN, USA; d Department of Medicine A, Haematology, Oncology and Pneumology, University Hospital Münster, Münster, Germany; e Department of Medicine A, University Hospital Muenster, Muenster, Germany; f Department of Respiratory Medicine and Thoracic Oncology, Foundation Mathias Spital, Rheine and Ibbenbueren, Germany; g Department of Thoracic Surgery, University Hospital Münster, Muenster, Germany; h Gerhard-Domagk-Institute of Pathology, University of Münster, Muenster, Germany; i Institute of Hygiene, University of Münster, Muenster, Germany

**Keywords:** Influenza B virus, human lung explants, lung organoids, cell tropism, innate immune response

## Abstract

The 2017/18 influenza season was characterized by unusual high numbers of severe infections and hospitalizations. Instead of influenza A viruses, this season was dominated by infections with influenza B viruses of the Yamagata lineage. While this IBV/Yam dominance was associated with a vaccine mismatch, a contribution of virus intrinsic features to the clinical severity of the infections was speculated. Here, we performed a molecular and phenotypic characterization of three IBV isolates from patients with severe flu symptoms in 2018 and compared it to an IBV/Yam isolate from 2016 using experimental models of increasing complexity, including human lung explants, lung organoids, and alveolar macrophages. Viral genome sequencing revealed the presence of clade but also isolate specific mutations in all viral genes, except NP, M1, and NEP. Comparative replication kinetics in different cell lines provided further evidence for improved replication fitness, tolerance towards higher temperatures, and the development of immune evasion mechanisms by the 2018 IBV isolates. Most importantly, immunohistochemistry of infected human lung explants revealed an impressively altered cell tropism, extending from AT2 to AT1 cells and macrophages. Finally, transcriptomics of infected human lung explants demonstrated significantly reduced amounts of type I and type III IFNs by the 2018 IBV isolate, supporting the existence of additional immune evasion mechanisms. Our results show that the severeness of the 2017/18 Flu season was not only the result of a vaccine mismatch but was also facilitated by improved adaptation of the circulating IBV strains to the environment of the human lower respiratory tract.

## Introduction

Seasonal influenza viruses (IV) of the types A and B (IAV and IBV) cause epidemic outbreaks of severe respiratory disease in humans accounting for up to 5 million laboratory-confirmed infections and 290-650,000 reported annual deaths worldwide [1]. Studies on influenza epidemiology were long primarily focused on IAV while the contribution of IBV was largely neglected. Since 2015, when IV surveillance was extended to include IBV, data revealed that IBV occasionally becomes the predominant circulating IV causing high numbers of hospitalized patients and therefore should be considered as a significant factor for the high global disease burden and economic costs caused by influenza epidemics [2].

Like IAV, IBV contains a partitioned genome with eight individual segments of single-stranded negative-sensed RNA that encodes for structural and non-structural proteins. In addition to the essential viral proteins of the viral polymerase (PB2, PB1, PA), the nucleoprotein (NP), the surface glycoproteins haemagglutinin (HA) and neuraminidase (NA), NS1/2 and the Matrixprotein1 (M1), the IBV genome encodes for the functionally unexplored protein NB instead of the proton channel Matrixprotein 2 (M2). So far, alternative proteins such as PB1-F2, PB1-N40, or PA-X for IAV have not been reported. Unlike IAV, which comprises 18 different Haemagglutinin (HA) and 11 Neuraminidase (NA) subtypes accounting for its broad species tropism in humans, animals, and birds, IBV is only divided into two lineages Victoria and Yamagata with distinct epidemic and evolutionary profiles in humans [3].

Evolutionary mechanisms of IBVs include nucleotide insertions and deletions, and inter – and intra-lineage reassortment. However, in general, IBVs exhibit much slower antigenic evolution compared to IAVs, and the Victoria lineage is characterized by higher rates of lineage change and antigenic drift than the Yamagata lineage [4,5]. Phylogenetic analyses by Langat and colleagues revealed that the divergence within Yamagata was not only driven by changes in the hemagglutinin (HA) and neuraminidase (NA) genes but also in the entire genome. Molecular clock phylogenetic analyses showed that genomic divergence started with the PB1 segment in 1993, followed by the PA segment in 1996, and then continued with the NP, PB2, HA, NA, NS1, and M1 segments between 2002 and 2003 [6]. By the 2017/18 influenza season, clade 2 had disappeared, leaving clade 3 as the dominant circulating clade within the Yamagata lineage. Virk et al reported that Yamagata viruses circulating between 2017–19 were characterized by mutations in the viral proteins NA, PB1, PA, and HA [7].

The sporadic epidemic dominance of IBV during the 2017/18 influenza season was demonstrated in Europe too, and this season was characterized by an unusually high number of severe infections caused by IBV of the Yamagata lineage. Most likely, this dominance was attributed to the absence of the B/Yamagata strain in the 2017 triple vaccine, which led to low vaccine efficacy [8–10]. However, intrinsic properties of the virus, mediated by mutations in viral proteins that increase pathogenicity, are suspected to have contributed to the high clinical severity of this influenza season [11]. This assumption is strongly supported by a recently published sequence analysis of more than 12,000 IBV genomes from 2008 to 2019, which revealed a major increase in the genetic diversity and evolution of Yamagata viruses. For Yamagata viruses in particular, antigenic drift of the neuraminidase was an important driver of epidemic activity [7].

IAV and IBV share the mechanisms of host transmission, many steps in viral replication, and induce similar disease symptoms. Generally, influenza viruses are transmitted to the upper respiratory tract via droplets or aerosols released from infected persons [12]. Viral replication begins in the respiratory tract epithelium after penetration of the columnar epithelial cells [13]. IBV infects human bronchi and a variety of cell types, including ciliated cells, club cells, goblet cells, and basal cells [14,15]. The expression of viral proteins, such as PB2 or PA interferes with the synthesis of cellular proteins and results in the induction of cell death mechanisms including apoptosis, necroptosis, pyroptosis [12,16,17]. Clinical symptoms such as inflammation and edema are frequently observed in the larynx, trachea, and bronchi. Histopathologically, tissue damage by vacuolization, cell loss, and sloughing of the ciliated columnar epithelial structure down to the basal cell layer are described in the columnar epithelium. Autopsy of fatal infections showed necrotic tracheobronchitis with ulceration and sloughing of the bronchial mucosa, bleeding foci, hyaline membrane formation, and a small number of neutrophils infiltration is observed [18].

A common complication of IAV and IBV infections is the induction of primary viral pneumonia when the virus is disseminated to the lower respiratory tract. Infection of the lungs leads to the loss of normal ciliated epithelial cells, submucosal hyperemia, focal bleeding areas, edema, immune cell infiltration of the alveolar space, alveolar edema, and bleeding and alveoli and hyaline membrane development. In addition to the damages mediated by the virus, influenza infection disrupts the clearance mechanisms of epithelial cells and increases the surface receptors that will facilitate the adhesion of bacteria to the epithelial surface, thus preparing the floor for secondary bacterial infections [19].

In 2018, patients with influenza B generally showed symptoms similar to those seen with influenza A infections, such as fever, cough, and fatigue. However, some differences were noted: vomiting and chronic kidney disease were more common in influenza B patients, while high-grade fever and respiratory distress were more common in influenza A patients. Hospitalization outcomes for influenza B included similar rates of pneumonia and respiratory failure compared with influenza A, but a significantly higher incidence of septic shock [20,21]. The 2017/18 Flu season was reported to be characterized by high hospitalization and mortality due to influenza and to last longer compared to previous years [22]. So far, detailed molecular studies to characterize the phenotype of Yamagata 2017/18 (clade 3A) viruses are rare and usually based on the application of traditional cell culture models, but a solid understanding of the mechanisms underlying their increased pathogenicity in the human lung is still largely missing.

Here, we characterized three clinical IBV isolates (Yamagata lineage) from the 2017/18 flu season in Germany by comparing their genome sequences, replication dynamics, and immune responses to an IBV isolate (Yamagata lineage) of the earlier season 2016 using replication models of increasing complexity including different cultured cells, primary human lung explants, and lung organoids as well as isolated alveolar macrophages. This sophisticated approach revealed that the IBV isolates from 2017/18 have evolved to acquire increased replicative fitness, tolerance to the higher temperatures of the lower respiratory tract, a broader alveolar cell tropism, and a reduced potential to induce protective type I and III responses in the human lung. Our results provide compelling evidence, that the clinical severity of the 2017/18 flu season was not only determined by the epidemiologic dominance of the circulating IBV/Yam viruses caused by low vaccine efficiency and high infection rates but also by a phenotypic evolution leading to improved infection of the lower respiratory tract. These new insights into the molecular mechanisms of IBV pathogenicity underscore the fundamental scientific value of primary human lung tissue models that advance the knowledge gained from traditional cell culture models and guide the way to improved clinical treatment strategies for respiratory virus infections.

## Material and methods

### Cells and viruses

Madin-Darby canine kidney (MDCK-II) cells and human lung adenocarcinoma cells (Calu-3) were cultivated in Dulbecco’s Minimum Essential Medium (DMEM, MERCK, Darmstadt, Germany) with 10% FBS (Capricorn, Ebsdorfergrund, Germany) and 1X Penicillin/Streptomycin (P/S) (Capricorn, Ebsdorfergrund, Germany) at 37°C and 5% CO_2._ Viruses from 2018 (GISAID: EPI_ISL_19135525, EPI_ISL_19135528, EPI_ISL_19135532) were isolated from clinical samples from patients with laboratory confirmed IBV infections presenting at the University Clinic Muenster with mild to severe symptoms as part of the clinical routine diagnostics (Table 1). Isolate B/Bayern/28/2016 (GISAID: EPI_ISL_19135514) was kindly provided by the National Reference Centre (NRZ) for Influenza at the Robert Koch Institute, Berlin.

**Table 1.** IBV isolates of Yamagata lineage used in this study.

Season	Lineage	Isolate	Abbreviation	Accession numbers (GISAID)
2016	Yamagata	B/Bayern/2016/28	B/16	EPI_ISL_19135514
2018	Yamagata	B/Münster/2018/337	B/18/337	EPI_ISL_19135525
		B/Münster/2018/338	B/18/338	EPI_ISL_19135528
		B/Münster/2018/341	B/18/341	EPI_ISL_19135532

### Viral RNA isolation and virus genome sequencing

Viral RNA was isolated from virus stocks using the QIAamp viral RNA mini kit (Qiagen, Hilden, Germany). Each vRNA segment was reverse transcribed using the Thermo Scientific Maxima™ Reverse Transcriptase (Thermo Fisher Scientific, Schwerte, Germany) and amplified by RT–PCR targeting the IBV genes with segment-specific primers (Table S1). Protocols for library preparation and sequencing were conducted as recommended by the manufacturer (Illumina). 1 ng of the PCR products was used for library preparation with the Nextera XT DNA Sample Preparation Kit (Illumina) and samples were paired-end sequenced with the 2x 250 bp MiSeq Reagent Kitv2 with an average insert size of 300 bp on a MiSeq instrument. After automatic demultiplexing on the MiSeq instrument, the resulting fastq files were mapped onto the Influenza B virus strain B/Germany/7058/2018 reference sequence (GenBank accession numbers: MK380427.1, MK380461.1, MK380393.1, MK380359.1, MK380325.1, MK380291.1, MK380257.1, MK380223.1) using the BWA mapping algorithm implemented in the Ridom SeqSphere + software (v7) with default parameters. The amino acid sequences of all three B/18 isolates were aligned to the sequence of the B/16/Bayern isolate using ClustalW. Clade specific and isolate-specific amino acid changes were identified using the Geneious software version 10.2.3.

### Phylogenetic analysis of the full-length HA and NA genes

The 93 HA and 90 NA sequences from Germany in 2018 were obtained from GISAID and analyzed in comparison with each other and the sequences of the strains B/16, B/18/337, B/18/338, and B/18/341. The GISAID ID numbers are listed in Table S3. The sequences were aligned with ClustalW 1.2.4_1 and aligned sequences were cleaned by Gblocks 0.91.1. Phylogenetic trees were constructed using the PhyML 3.3_1 maximum likelihood method (500 bootstrap replicates) provided by the NGPhylogeny.fr platform. Additionally, 114 HA and 107 NA sequences from Europe in 2016, and 176 HA and 161 NA sequences from Europe in 2014 were aligned with the B/16, B/18/337, B/18/338, and B/18/341 HA and NA sequences. The nucleotide distances of HA and NA sequences of the isolates used in this study with isolates from the previous seasons were estimated by using Geneious Prime® 2024.0.5. [23].

### IC_50_ determination of the neuraminidase inhibitor Oseltamivir (OST)

Calu-3 cells were infected with a multiplicity of infection (MOI) 0.01 for 30 min at 33°C. Cells were washed with iPBS (PBS containing 1X P/S), 0.2% BSA (Sigma-Aldrich, St. Louis, USA), 0.01% MgCl_2_ (Carl Roth, Karlsruhe, Germany), 0.01% CaCl_2_ (Carl Roth, Karlsruhe, Germany) and medium (DMEM high glucose supplied with 1X P/S, 0.2% BSA, 0.01% MgCl_2_, 0.01% CaCl_2_, 0.02% TPCK-Trypsin (Sigma-Aldrich, St. Louis, USA)) containing increasing amounts of OST (0-1000 nM) was added for 48 hours. Virus titers were determined by plaque assay. The IC_50_ was determined using the “log(inhibitor) vs. normalized response – Variable slope (four parameters)” equation in GraphPad Prism 8.0.1 software.

### Human lung explants

Tumor-free human lung tissue was donated from patients undergoing surgical lung resection at the Department of Thoracic Surgery at the University Clinic Muenster and the Department for Thoracic Oncology of the Foundation Mathias Spital, Rheine and Ibbenbueren. All donors gave their written consent to donate explant tissues for scientific use. Ethical approval was given by the ethical council of the Ärztekammer Westfalen-Lippe (AZ 2016-265-f-S). Tissues were transferred into cold RPMI directly after surgery and kept chilled at 4°C until further use.

### Isolation of alveolar macrophages from human lung explants

Human lung tissue was injected with Hanks´ Balanced Salt Solution (HBSS, Thermo Fisher Scientific, Waltham, USA) to flush out cells. Red Blood Cell Lysis Solution (RBC solution, Miltenyi Biotec, Bergisch Gladbach, Germany) was added to remove erythrocytes from the cell solution. The remaining cells were seeded into wells of a culture plate in macrophage growth media (RPMI 1640 supplemented with 2% FCS, 1% L-glutamine (Sigma-Aldrich, St. Louis, USA) and 1X P/S). After 2 hours media was replaced with fresh media and alveolar macrophages were incubated 4 days before infection.

### Virus infection of cells

Virus stocks of the IBV isolates (Table 1) were prepared in MDCK-II cells at 33°C for 48 h. Infections were performed for 30 min in iPBS and incubated with DMEM high glucose supplied with 1X P/S, 0.2% BSA, 0.01% MgCl_2_, 0.01% CaCl_2_, 0.02% TPCK-Trypsin (Sigma-Aldrich, St. Louis, USA) at 33°C and 5% CO_2,_ until 72 hpi. For determination of growth curves, MDCK-II and Calu-3 cells were seeded to 70% confluency and infected at a multiplicity of MOI of 0.01 for 30 min at 33 and 37°C at 5%CO_2_. Supernatants were collected at 8, 24, and 48 hpi.

### Virus infection of human lung tissue and macrophages

100 mg pieces of human lung tissue were infected with 2 × 10^6^ PFU virus for 1 h at 33°C (and 37°C wherever indicated). The virus was removed and replaced with fresh medium (RPMI 1640 supplemented with 1% P/S, 1% L-glutamine (Sigma-Aldrich, St. Louis, USA), and 0.3 Bovine Serum Albumin (BSA, Sigma-Aldrich, St. Louis, USA)). Supernatants were collected at 1, 24, and 48 h for virus titration. Tissues were fixed with 4% paraformaldehyde (PFA, Merck, Darmstadt, Germany) diluted in PBS for 16 hpi to perform immunohistology staining [24] or harvested and put in RNAlater™ (Thermo Fisher Scientific, Waltham, USA) until total RNA isolation for transcriptome analysis [25].

Isolated alveolar macrophages were seeded into 12 well plates with a cell density of 1 × 10^6^ cells/well. Cells were washed with iPBS and infected at an MOI of 1 for 1 h at 33 and 37°C with 5% CO_2_. Supernatants were collected for titration at 1, 24, 48, and 72 hpi.

### Plaque assay

Supernatants were serially diluted 1:10 in iPBS. MDCK-II cells were washed with iPBS and incubated with 250 μl of the diluted supernatants at 33°C and 5% CO_2_. After 30 min virus was aspirated and freshly prepared overlay medium (9.9% 10x MEM (Thermo Fisher Scientific, Waltham, USA), 0.2% BSA, 1X P/S, 0.3% DEAE-Dextran (Sigma-Aldrich, St. Louis, USA) and 1.5% NaHCO_3_ (Thermo Fisher Scientific, Waltham, USA), 0.9% Agar (Thermo Fisher Scientific, Waltham, USA), 0.01% MgCl_2_, 0.01% CaCl_2_, 0.02% TPCK-Trypsin) was added. Plates were incubated at 33°C and 5% CO_2_ for 5 days until plaques were countable.

### Immunohistochemistry

Fixed tissues were dehydrated with a series of increasing isopropyl alcohol (70%−100%), embedded in paraffin, and cut into 4 µm sections. Tissue sections were deparaffinized in xylene followed by rehydration in descending isopropanol dilutions. Antigen retrieval was performed in citrate buffer (Abcam, Cambridge, UK) for 30 min. Sections were permeabilized and blocked with blocking buffer (PBS with 10% FBS and 0,1% Triton X100) for 30 min followed by incubation with IBV nucleoprotein-specific mouse monoclonal antibody (orb69084, Biorbyt, Cambridge, UK, 1:10000 in blocking buffer) antibody, Anti-human podoplanin (PDPN) antibody (1:500 in blocking buffer, ab128994, Abcam, Cambridge, UK) and Anti prosurfactant protein C (SFTPC) antibody (1:2000 in blocking buffer, AB3786, Sigma, Darmstadt) for 1 h and biotinylated horse anti-mouse IgG (1:500 in blocking buffer, BA-2000-1.5, Vector Labs, Burlingame, CA) or biotinylated goat anti-rabbit IgG (1:500 in blocking buffer, BA-1000, Vector Labs, Burlingame, CA) for 30 min. After incubation with VECTASTAIN® ABC-AP kit (Vector Laboratories, Burlingame, CA, USA) for 30 min, the sections were developed with Vector® Red Substrate Kit (Vector Laboratories, Burlingame, CA, USA). After nuclear staining with Haematoxylin (Roth, Karlsruhe, Germany) the sections were blued with tap water, dehydrated in 70%, 96%, and 100% isopropyl alcohol, cleaned with xylene and mounted with RotiHistokit (Roth, Karlsruhe, Germany). Slides were inspected using the Axiovert 200M microscope and images were taken via the Axiocam ICc1 camera using the 20X and 100X objectives and processed via the AxioVision Rel 4.8 software.

### Bulk RNAseq and data analysis

Infected lung tissues in RNALater were transferred to Lysing Matrix A tubes (MP Biomedicals) with 500 μl RLT lysis buffer supplemented with 5 µl β-mercaptoethanol and shredded using FastPrep®−24 (MP Biomedicals). For total RNA isolation, the RNeasy Plus Mini Kit (Qiagen) was used according to the manufacturer`s instructions.

RNA integrity was verified using Agilent Technologies 2100 Bioanalyzer (Agilent Technologies, Germany). Mature mRNAs were purified from 1 μg of total RNA using Dynabeads mRNA DIRECT Micro Kit (ThermoFisher, USA), and sequencing libraries were prepared using NEBNext Ultra II Directional RNA Library Prep Kit (New England BioLabs, USA), following manufacturer’s protocols. Libraries were sequenced on Illumina NovaSeq 6000 using NovaSeq 6000 S1 Reagent Kit (300 cycles, paired end run 2 × 111 Nu) with an average of 6.4 × 10^7^ reads per sample.

Reads from RNAseq were quality-checked with FastQC (version 0.11.9, [26]) and trimmed using Trimgalore (version 0.6.7, [27]) with default settings. Trimmed reads were mapped to the human genome (ENSMBL hg38 release 100) using STAR (version 2.5.2b, [28]) with default settings. Mapped reads were counted at the gene level using RsubRead (version 1.32.4, [29]).

Virus sequences were identified by mapping to the Influenza B virus genome (NCBI: txid11520|B/Germany/7058/2018). Expression data was analyzed and visualized using the R software (version 4.2.1, [30]) and RStudio (version 2022.07.2, (RStudio)). Human genes were annotated using biomaRt package (version 2.52.0, annotation GRCh38.p12, [31]). Raw counts of host genes and viral-genes were combined, normalized, and log_2_-transformed using rlogTransformation function from DESeq2 package (version 2.7.9a, [32]). An increment was added to the normalized values to make all values positive. For identification of differentially expressed genes (DEGs), DESeq2 package was used with the following model and batch correction for donors: design = ∼donor + group; groups: “Mock_48hpi”, “FluB_337_24hpi”, “FluB_337_48hpi”, “FluB_Bay_24hpi”, “FluB_Bay_48hpi”; donors: HULU_285, HULU_288, HULU_294. DEGs were identified using thresholds of < 0.05 for adjusted *p*-value and >1.5-fold difference (|log2| > 0.5849625) in expression levels. Volcano plots and heatmaps were generated using the EnhancedVolcano and pheatmap functions respectively within ggplot2 package using default parameters as described previously [33]. Gene Ontology (GO) analysis was performed with clusterProfiler [34] function using the biological processes (BP) module and visualized using ggplot2.

### Statistical analyses

Statistical analysis was performed with GraphPad Prism Version 8.0.1 (GraphPad Software Inc., San Diego CA) unless otherwise specified. Replication kinetics with repeated measures (RM) were compared by two-way ANOVA for multiple comparisons. IC_50_ values were determined by non-linear regression. Statistical significance was determined between B/16 and B/18 at a given time point using one-way ANOVA followed by Dunnett's multiple comparison test.

## Results

### Phylogenetic analysis of viral isolates from three clinical IBV cases demonstrates ongoing evolution

**Figure 1.**
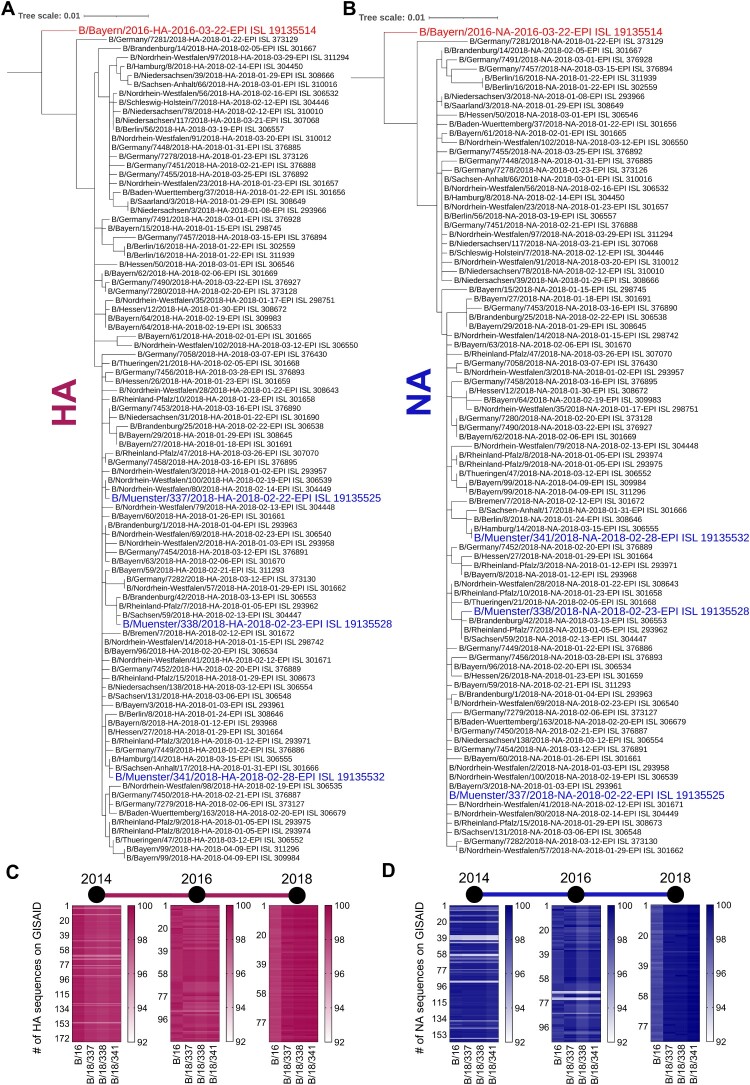
Phylogenetic trees based on HA (A), and NA (B) nucleotide sequences of IBV isolates from 2018, including B/16 isolate. Nucleotide sequences were analyzed for phylogenetic relationships by the distance method using NGPhylogeny platform. B/18 Muenster isolates were highlighted in blue and B/16 in red. The nucleotide sequence similarity in percentage (right columns) of HA (C) and NA (D) was estimated for all IBV sequences available (y-axis) on GISAID for the years 2014 in Germany (176 for HA, 161 for NA), 2016 in Germany (114 for HA, 107 for NA) and 2018 in Europe (93 for HA, 90 for NA). The nucleotide sequence similarity matrix was generated by using Geneious Prime® 2024.0.5.

To gain increased knowledge on the molecular mechanisms underlying the remarkable pathogenicity of IBV infections during the 2017/18 flu season three IBV isolates and one isolate from 2016, all acquired from patients with mild to severe flu-like symptoms, were submitted to full genome sequencing (Table S2). A maximum likelihood (ML) phylogenetic analysis of the HA (Figure 1(A)) and NA (Figure 1(B)) sequences with available sequences from Germany and Europe revealed that the B/18/337, B/18/338, and B/18/341 isolates cluster well within the other 2018 IBV viruses while the IBV strain from 2016 (B/16) remains separated, demonstrating their evolutionary distance. Heat maps displaying the percentages of similarity between the B/16, B/18/337, B/18/338, and B/18/341 HA (Figure 1(C)) and NA (Figure 1(D)) sequences with sequences obtained from GISAID for the depicted years show that the HA and NA sequences of the three B/18 isolates share the highest similarity (99%) with the 2018 isolates and B/16 isolate with the 2016 isolates. Altogether, these results demonstrate, that the four isolates B/16, B/18/337, B/18/338, and B/18/341 are good representatives of the circulating IBV strains in the 2018 season.

### Mutations in the neuraminidase have no effect on the sensitivity towards oseltamivir

Comparing the amino acid sequences of all viral proteins between B/16 with the three B/18 strains identified 31 mutations in isolate B/18/337, 33 amino acid changes in B/18/338, and 31 amino acid changes in B/18/341. While the majority of these mutations were present in all three B/18 isolates and reflected characteristic mutations of the 2017–2018 Clade 3A viruses (Table 2, red) [7], also several isolate-specific mutations that were not described before were identified in our study (Table 2, blue).

**Table 2.** Mutations in proteins of the B/18 isolates from this study compared to the B/16 isolate. Bold numbers refer to characteristic mutations of the 2017-2018 Clade 3A [7]. Italic numbers refer to mutations that are specific for the indicated virus isolate.

Proteins	B/18/337	B/18/338	B/18/341
PB1	**A591S**	*V57I*, **A591S**	*V164I*, *R327K*, **A591S**
PB2	R115K, Q354H, N442S, *E467G*	R115K, Q354H, N442S	R115K, Q354H, N442S
PA	**M326V**, *N395K*, K417R, *I485V*, S547G, I594V	**M326V**, K417R, S547G	K417R, S547G, I594V
HA	R226K, L453I	*P46Q*, R226K, *K268S*, L453I	*N211S*, R226K, L453I
NA	V49M, T50M, R65H, M81T, V120I, **I171M**, **N342K**, K373Q, I401V, S402P	*L38P*, V49M, T50M, R65H, *A67T*, *L74P*, M81T, V120I, **I171M**, **N342K**, K373Q, I401V, S402P	V49M, T50M, R65H, M81T, *T106I*, V120I, **I171M**, **N342K**, K373Q, I401V, S402P
NB	V21A, S51N, N52D, S63P, I92L	V21A, S51N, N52D, S63P,*C69H*, I92L	V21A, S51N, N52D, S63P, I92L
BM2	I21M	I21M	I21M
NS1	K110E, K176R	*K106R*, K110E, K176R	K110E, K176R
NP	None		
M1	None		
NEB	None		

**Figure 2.**
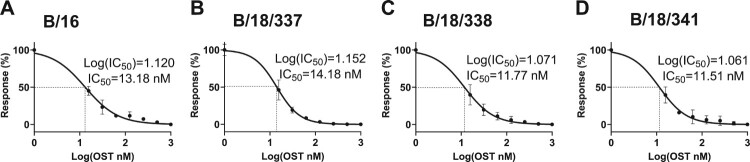
Determination of IC_50_ values of Oseltamivir (OST) for the inhibition of viral replication. Calu-3 cells were infected with the indicated IBV viruses at MOI 0,01. Virus titers were determined after 48 hpi by plaque assay. IC_50_ values were calculated using GraphPad 8.0.1(n ≥ 3)

Interestingly, the NA protein contained the highest number of mutations in all three B/18 isolates. Several of them were located within the NA head domain, however, none of them was reported to be associated with drug resistance against NA-targeting antivirals. To assess the possibility that these mutations confer resistance to the clinically used NA inhibitor oseltamivir (OST) the IC_50_ values of all three B/18 isolates and the B/16 isolate were determined and compared. IC_50_ values of OST were similar for the three B/18 isolates as well as the B/16 isolate with 13.18 nM for B/16, 14.18 nM for B/18/337, 11.77 nM for B/18/338, 11.51 nM for B/18/341, demonstrating no changes in the susceptibility of the isolates against OST treatment (Figure 2).

### B/18 isolates demonstrate altered growth phenotypes in cell culture and human lung explants

**Figure 3.**
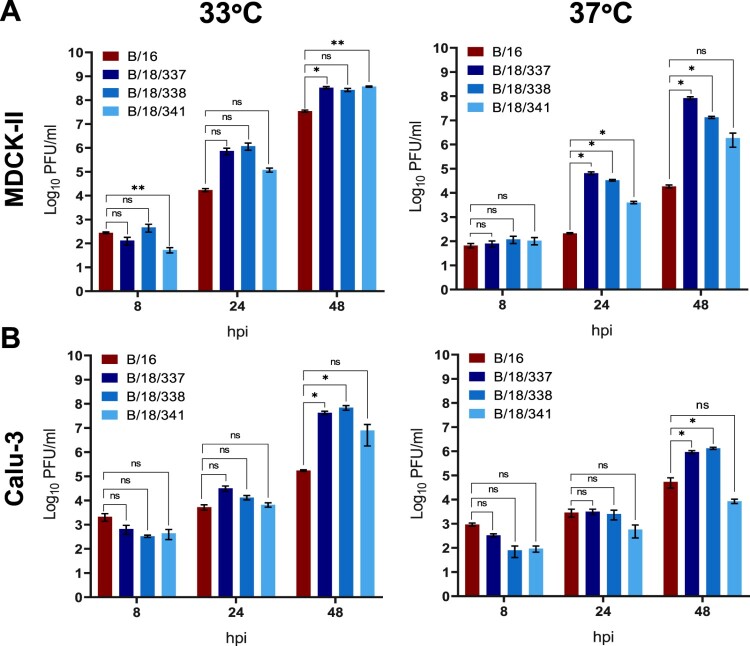
Virus replication kinetics of B/16, B/18/337, B/18/338 and B/18/341 in MDCK-II cells (A), Calu-3 (B) cell lines. Supernatants were collected 8, 24 and 48 hpi and virus titers were determined by classical plaque assay. Virus titers are shown as mean titers ± SD of three biological replicates and the significance test was done by two-way ANOVA and Dunnett’s multiple comparison tests against B/16 (ns *p* > 0.05, * *p* ≤ 0.05, ** *p* ≤ 0.01, *** *p* ≤ 0.001 and **** ≤ 0.0001)

According to the mutations identified within the glycoproteins and the three polymerase proteins, adaptations in viral fitness and infectivity phenotypes were analyzed. First, the viral replication kinetics were determined in the immune-incompetent cell line MDCK-II which is commonly used for vaccine production due to a deficient interferon-induced antiviral activity against influenza viruses [35–37]. At 33°C degrees, the temperature of the upper respiratory tract, all four isolates replicated efficiently to titers above 10^7^ PFU/ml at 48 h post-infection (hpi). However, while the 2016 isolate reached 1.7 × 10^4^ PFU/ml at 24 hpi and 3.5 × 10^7^ PFU/ml at 48 hpi, the two B/18 isolates B/18/337 and B/18/338 demonstrated improved replication reaching titers of 7.5 × 10^5^ and 1.2 × 10^6^ PFU/ml at 24 hpi, 3.3 × 10^8^ and 7.7 × 10^8^ PFU/ml at 48 hpi, respectively (Figure 3(A) left panel). This difference in replication fitness was further increased at 37°C, which resembles the temperature in the lower respiratory tract. Elevated temperatures resulted in strongly reduced replication of B/16 reaching only 1.9 × 10^4^ PFU/ml at 48 hpi. In contrast, titers of the isolates B/18/337 and B/18/338, and also B/18/341, albeit to a lesser extent, were only mildly reduced providing evidence for the development of a remarkable tolerance to higher temperatures by the 2018 isolates (Figure 3(A) right panel).

In the next step, immune-competent Calu-3 cells were employed. At 33°C replication of B/16 reached only 1.7 × 10^5^ PFU/ml at 48 hpi (Figure 3(B)), representing a reduction by two log steps compared to MDCK-II cells, which was likely mediated by the intact type I IFN response. Likewise, replication of the three B/18 isolates was reduced by approx. two log steps at 24 hpi. However, this reduction was partially compensated at 48 hpi, suggesting potent mechanisms to escape from the IFN induced antiviral response (Figure 3(B), left panel). Interestingly, the temperature dependent attenuation of B/16 at 37°C was less pronounced in Calu-3 cells compared to MDCK-II cells at 24 hpi, however, titers did not increase within the following 12 hours, supporting the growth defect at higher temperatures (Figure 2(B), right panel). Replication of the 2018 isolates was differentially affected and demonstrated isolate specific phenotypes. While isolates B/18/337 and B/18/338 were attenuated by one log step at 48 hpi reaching titers of 9.3 × 10^6^ and 1.3 × 10^6^ PFU/ml, replication of B/18/341 was more strongly attenuated only reaching titers of 8.7 × 10^3^ PFU/ml at 48 hpi (Figure 3(B), right panel). This growth defect could be mediated by differences in the amino acid sequence in the polymerase or glycoproteins since isolate B/18/341 differs by several positions to the other two B/18 isolates (Table 2).

### B/18 isolates demonstrate broader cell tropism in human lung explants

**Figure 4.**
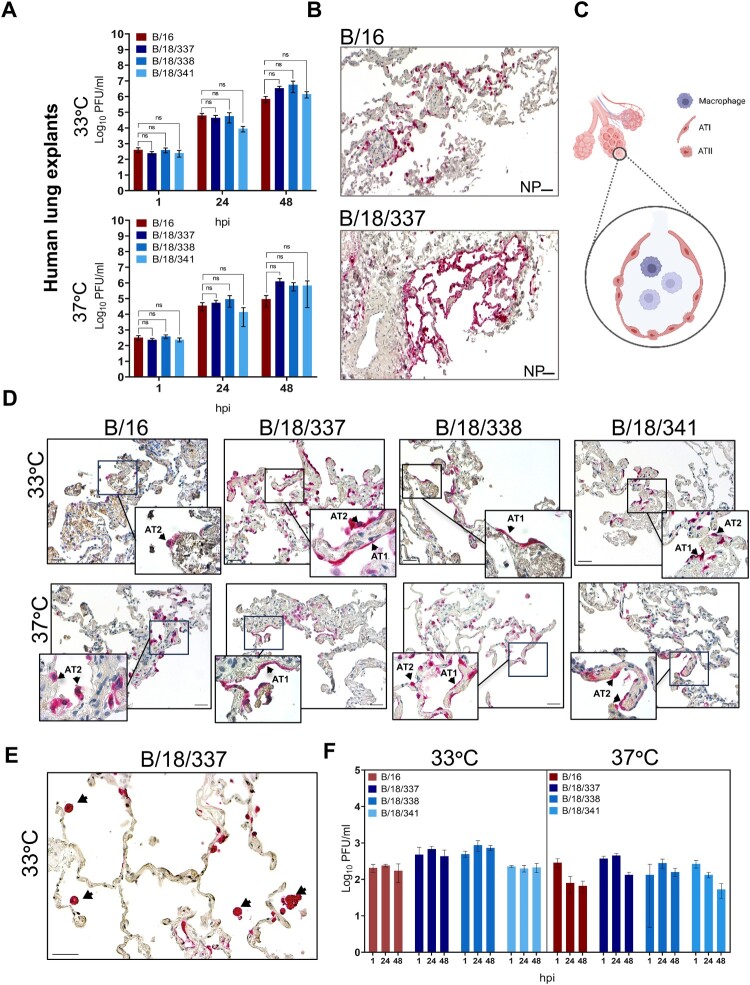
Virus replication kinetics of B/16, B/18/337, B/18/338, and B/18/341 in human lung explants (n ≥ 6) (A). Supernatants were collected at 1, 24 and 48 hpi, and virus titers were determined by classical plaque assay. Virus titers were shown as mean titers ± SD of three biological replicates and the significance test was done by two-way ANOVA and Dunnett’s multiple comparison test against B/16 (ns *p* > 0.05, * *p* ≤ 0.05, ** *p* ≤ 0.01, *** *p* ≤ 0.001 and **** ≤ 0.0001). The immunohistochemical staining of the nucleoprotein of B/16 and B/18/337 isolates (pink) in virus-infected explants at 33°C with lower objective magnification (20X) (B). The figure represents alveolar structure and different cell types (C) Immunohistochemical staining of nucleoprotein of all B/16 and B/18 isolates (pink) in virus-infected explants at 33°C and 37°C (D) at 33°C and 37°C (n ≥ 3). Immunohistochemical stainings of the nucleoprotein of isolate B/18/337 (pink) in virus-infected explants at 33°C (arrows indicate NP-positive macrophages) (E), Virus replication kinetics of B/18/337, B/18/338, and B/18/341 in macrophages isolated from human lung (n ≥ 3) at 33°C and 37°C (F). Scale bars = 50µm

Finally, we investigated the replication fitness of all four IBV isolates in primary human lung tissue following *ex vivo* infection. Lung explants were derived from the lower respiratory tract, resembling the alveolar compartment of the lung. Figure 4(A) shows that all isolates replicated in human alveolar lung tissue and reached titers between 10^5^–10^6^ PFU/ml at 48 hpi, albeit the titers of B/16 were slightly lower (7.2 × 10^5^) compared to the 2018 isolates (approx. 3.5 × 10^6^) at 33°C (Figure 4(A), upper panel). Interestingly, while the 2018 isolates were not affected by the higher temperature as demonstrated by continuously increasing viral titers over 48 h, replication of B/16 at 37°C was nearly halted after 24 h (Figure 4(A), lower panel) suggesting a temperature dependent decrease in viral replication.

Immunohistochemical staining of infected cells 48 hpi using an antibody specific to the viral nucleoprotein (NP) revealed marked differences in virus dissemination and cell tropism between B/16/Bayern and the B/18 isolates, most pronounced by isolate B/18/337 (Figure 4(B)). The pulmonary alveolar epithelium consists of type 1 (AT1) and type 2 (AT2) alveolar epithelial cells. AT2 cells, the known primary target cells for IV [38] are cuboidal cells and intersperse the thin alveolar walls that are majorly formed by the long and flat AT1 cells (Figure 4(C), Figure S1). A more detailed analysis of the infected cells demonstrated that infection with B/16 at 33°C and 37°C was restricted to AT2 cells (Figure 4(B), upper panel). In contrast, NP staining was additionally detected in AT1 cells in lungs individually infected with the three B/18 isolates at 33°C and 37°C (Figure 4(D), upper and lower panel). To determine if the increased titer of B/18/337 at 48 hpi is due to better replication in AT2 cells, AT2-derived lung organoids were infected with B/16 and the B/18 isolate B/18/337. However, this resulted in the same titers at 48 hpi for both viruses (Figure S2), suggesting that the higher viral titers of B/18/337 in lung tissue at 48 h are rather a consequence of infection of AT1 cells.

In addition to infection of cells from the lung epithelium, NP-positive alveolar macrophages were frequently observed after *ex vivo* infection (Figure 4(E)). This suggested that macrophages can also be infected and thereby contribute to viral replication and lung damage due to compromised protective functions [39]. To analyse whether macrophages are productively infected, we purified alveolar macrophages from lung tissue and infected them with B/16 and B/18/337. Plaque assays from the supernatants revealed that the titers remained on the level of the virus input (titer at 1 hpi) at 24 and 48 h, indicating that the infections were abortive and did not result in the production of new infectious virus particles as described for seasonal IAV and IBV (Figure 4(F)). This further supports, that the spread of B/18 to AT1 cells is responsible for the increased titers of the B/18 isolates in the lung model.

### Isolate B/18/337 induced a weaker antiviral response mediated by decreased interferon induction in human lung explants

**Figure 5.**
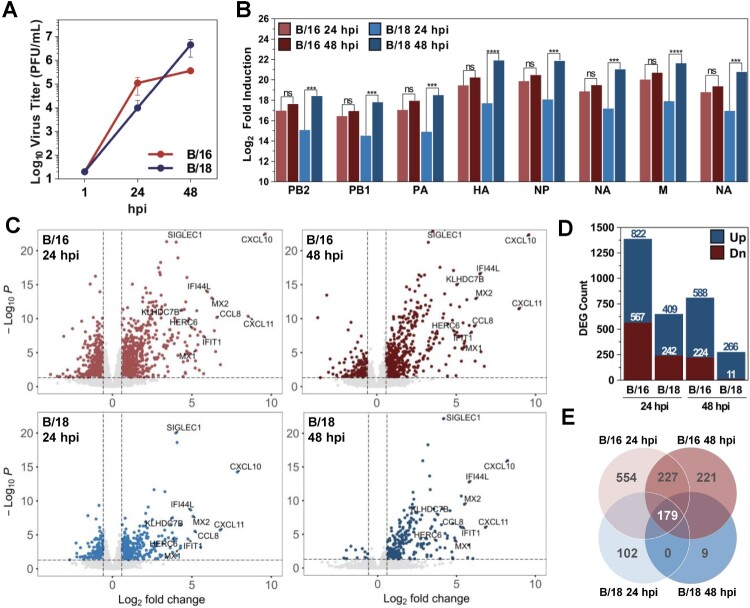
Bulk RNAseq of primary human lung tissues reveals a weaker immune response to infection with B/18/337 isolate compared to B/16 isolate. Replication kinetics of B/16 and B/18/337 isolates in primary human lung tissues. Virus titers in the supernatants collected at 24 and 48 hpi were quantified by standard plaque titration on MDCK-II cells (A). Comparison of viral mRNA levels at 24 and 48 hpi for the depicted segments for B/16 (red) and B/18/337 (blue). Data are represented as log_2_ fold changes over uninfected control. Statistical significance was determined between 24 and 48 hpi for each virus using one-way ANOVA followed by Dunnett's multiple comparison test. *p < 0.05; **p < 0.01, ***p < 0.001 (B). Volcano plots of genes differentially expressed after infection by B/16 (upper panels) and B/18/337 (lower panels) at 24 hpi (left panels) and 48 hpi (right panels). DEGs (coloured) were identified by using an adjusted p.value <0.05 and an absolute fold change of 1.5 (log_2_ = 0.58496254) (C). Barplot depicting the number of up (blue) and down-regulated (red) DEGs for each condition (D). Venn diagram depicting the numbers of DEGs induced by each virus at a given timepoint compared to uninfected controls; 179 DEGs were shared by both viruses (E).

Increased replication in the immunocompetent cell line Calu-3 (Figure 3(B)) suggested the evolution of improved host immune antagonistic properties by the B/18 isolates. This could be facilitated by the identified mutations in the viral polymerase or the viral NS1 gene products, both of which are known as immune antagonists for IAV and IBV [40,41]. Transcriptome analysis by bulk RNA sequencing (RNAseq) of *ex vivo* infected lung tissue was performed to investigate whether the effect in Calu-3 cells could be recapitulated in the natural environment of the human lung. Therefore, lung tissue from three individual donors was infected at 33°C with isolates B/16 or B/18/337 as described before, the extracellular viral titers were measured at 24 and 48 hpi, and the infected lung tissues were harvested for transcriptomic analysis. Measured virus titers in the supernatant of the infected lungs (Figure 5(A)), as well as the expression levels of viral mRNAs (Figure 5(B)), indicated that B/18, compared to B/16, replicated slower in the first 24 hpi reaching titers of 3 × 10^4^ PFU/ml but faster in the proceeding 24 h reaching 1.35 × 10^7^ PFU/ml at 48 hpi. In contrast, B/16 already reached 3.33 × 10^5^ PFU/ml at 24 hpi but then continued with a limited growth dynamic to 1.09 × 10^6^ PFU/ml at 48 hpi.

**Figure 6.**
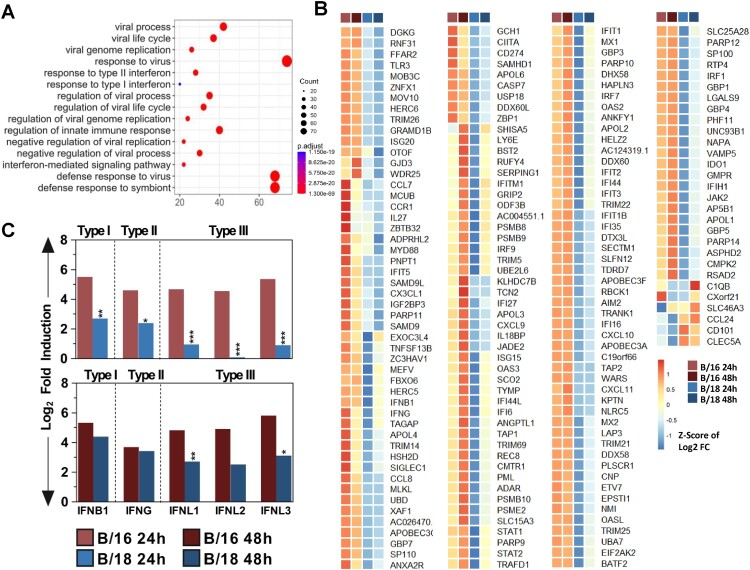
Differential expression analysis of DEGs commonly induced by B/16 and B/18/337 isolates in primary human lung tissue. Gene Ontology (GO) analysis of the 179 core DEGs using ClusterProfiler for Biological Processes (BP); Top 15 biological processes were visualized in terms of gene counts and adjusted p-values (A). Heatmap of the 179 core DEGs for the two virus isolates at 24 and 48 hpi. Log_2_ fold changes (over uninfected control) were scaled row-wise for each gene across the different conditions and expressed as Z-scores (B). Comparison of differential induction of selected IFN genes across different conditions (C). Data represented as log_2_ fold changes (over uninfected control). Statistical significance was determined between B/16 and B/18/337 at a given timepoint using one-way ANOVA followed by Dunnett's multiple comparison test. *p < 0.05; **p < 0.01, ***p < 0.001.

Principle component analysis of the normalized reads revealed a dominant donor effect determined by the individual genetic background of the tissue donors (Figure S4, right panels, and Table S5) obscuring the analysis of treatment-dependent changes in the transcriptomes. Therefore, the raw data were further normalized using the donor-correction package in R, which resulted in a clear treatment-dependent clustering (Figure S4, left panels). Analysis of the induced host responses showed that B/16, compared to B/18/337, infection resulted in a higher number of up-and downregulated differentially expressed host genes (DEGs) at 24 and 48 hpi (Figure 5(C) upper panels, and D). In contrast, despite the higher titers at 48 h, the response induced by B/18/337 was weaker and characterized by the induction of lower numbers of DEGs (Figure 5(C) lower panels, and D). In-depth analysis revealed, that 179 genes were regulated by both viruses compared to the uninfected control (Figure 5(E)). Gene ontology demonstrated that these 179 DEGs were associated with viral processes and the interferon response to viral infections (Figure 6(A)). Comparing gene expression levels of the 179 shared genes in a heat map demonstrated that infection with B/18/337 induced lower expression levels of these genes than infection with B/16, at both time points, despite higher viral titers of B/18 at 48 hpi (Figure 6(B)). This included important IFN-induced viral restriction factors such as *MX1, OAS1, ISG15.* Furthermore, in accordance with the weaker induction of interferon-stimulated genes (ISGs), B/18/337 induced significantly lower expression levels of the type I IFN *IFNB1*, type II IFN *IFNG,* and III IFNs *IFNL1, IFNL2, and IFNL3* compared to mock-treated controls at 24 h and for some genes also at 48 hpi (Figure 6(C)). Taken together, these data support our previous hypothesis, that B/18 have evolved improved mechanisms to counteract the induction of IFN-dependent antiviral responses.

## Discussion

IBV represents a relevant public health problem causing severe respiratory infections. Since 2015, IBV has demonstrated increasing pathogenicity and recurring seasonal dominance [37,42]. Especially in the 2017/18 influenza season, the sharp increase in the number of patients with influenza-like illness (ILI) suggested that the circulating virus may have escaped herd immunity and mutated to achieve higher infectivity [43]. The results from our study provide missing experimental evidence demonstrating that not only low vaccine efficacy but also a phenotypic evolution of IBV/Yam facilitated improved infection of the lower respiratory tract and thereby likely contributed to the severity of the infections causing unusual high hospitalization rates.

Viral genome sequencing allowed us to perform a phylogenetic analysis of the viral glycoproteins HA and NA revealing high similarity to other available sequences from the same season but clear divergence from the HA and NA sequence of an IBV/Yam isolate from 2016 (Figure 1). It is well described that several HA antigenic regions play a major role in receptor specificity. For IBV these regions are located at residues 116–137 residues (120 loops), residues 141–150 (150 loops), residues 162–167 (160 loops), and residues 193–202 (190 loops) which contain the receptor binding domain (RBD). Although IBV is generally considered to be more genetically stable compared to IAV our study identified a high number of mutations in the proteins of the three B/18 isolates compared to the B/16 isolate [44]. Protein alignments revealed two mutations at position R226K and L453I in the head and stalk domains in the HA of all three B/18 isolates. In addition, HA of B/18/338 contained the unique mutations P46Q and K268S, and B/18/341 harbored the additional mutation N211S, which were all not described before (Table 2 and Figure S1). Since none of these mutations is precisely located in one of the described loops nor the RBD, predictions on a possible role in receptor binding and cell tropism are difficult to make and will require additional experimental investigations.

With a 10–13 difference to B/16, the NA of the B/18 isolates harbored the highest number of mutations among all viral proteins. Eight mutations to B/16 were found at positions V49M, T50M, F65H, M81 T, V120I, K373Q, I401V and V402P in all three B/18 isolates. In addition, the previously described clade 3A-specific mutations I171M and N342K were also found. Functionally, Chen et al. could show that N342K increases NA activity in a mouse model and speculated that this mutation contributes to the increased contagiousness of clade 3A IBV [45]. B/18/338 and B/18/341 NA harboured unique mutations at L38P, A67T and L74P or T106I, respectively. So far, we cannot provide experimental data on the biological and functional relevance of the identified mutations in HA, NA, or other viral proteins identified in our study nor establish a functional link to the described phenotypic changes as we are lacking suitable reverse genetic systems. However, the combination of our data from different experimental models still provides strong indications for genotype-to-phenotype mechanisms. As different viral proteins are involved in virulence enhancing processes, we speculate that rather a combination of mutations in different viral proteins could be involved in mediating our reported phenotypic changes. For example, it was shown that not only the NS1 protein [41,46] but also some specific motifs in PB1 and PA subunits as well as NP of IAV are involved in interferon antagonism [47]. In line with this, Schreiber et al. described that amino acid A523 in the IBV PB1 thumb domain confers IFN-antagonistic properties [48]. Intriguingly, one of the mutations, A591S, that we identified in PB1 is also located in the thumb domain. We therefore assume that the mutations in the viral polymerase (A591S, R115K, Q354H, N442S) or the NS1 protein C-terminal region (K110E and K176R) could be associated with the higher viral fitness and reduced induction of IFNs in human lung tissue by the B/18/337 virus. Furthermore, Donelan et al. showed that deletion of the NS1 protein caused replication impairments in systems with IFN deficiency, indicating that NS1 may have additional roles other than IFN antagonism [41,49].

While the severity of influenza epidemics is commonly retrospectively correlated to the efficacy of the preceding vaccine and the resulting level of protective immunity, our data demonstrate that alterations in the viral phenotype can strongly influence the pathophysiology of viral infections and should be considered as important determinants for seasonal severity. Changes in cell tropism resistance to higher temperature and enhanced antagonism of IFN induction [50–53] and signaling have also been reported for other viruses and we are convinced that our results are therefore not only relevant to the 2018 IBV/Yam isolates but also shed light on the general mechanism that leads to the described pathophysiological consequences and thereby can be of relevance also to other influenza or respiratory viruses.

While the results of our study provide compelling experimental evidence for the impact of phenotypic evolution of IBV on its high virulence in 2018, our study also has several limitations. Due to the lack of a reverse genetics system for the 2016 and 2018 isolates we were not able to unravel which mutations in the 2018 IBV genome are causative for the described alterations in the viral phenotype. In addition, including more IBV strains in the analysis would be helpful to further strengthen the general character of our results. Since the COVID-19 pandemic, no confirmed infections with IBV of the Yamagata lineage were reported, leading to the impression that it has become extinct. In addition, the lack of animal reservoirs limits the possibilities for the resurgence of IBV from unexpected hosts. However, this impression may be obscured since IBV mostly presents with mild symptoms. Hence, the low hospital admission rates of IBV-infected patients may make it difficult to bring positive cases to the public attention. Another aspect that may cause the impression of IBV/Yam eradication is the lack of differentiation of IBV infections caused by the Yamagata or Victoria lineage, which is also represented in the WHO surveillance data [54]. This lack of lineage differentiation may be caused by the absence of IBV/Yam infections or the lack of testing for these IBV/Yam infections because they are not expected to occur. The latter could lead to unrecognized IBV/Yam cases. Additionally, reassortment between the two IBV linages is feasible and may lead to the preservation of the IBV/Yam genetic material supporting its re-emergence. While this has also happened to other influenza strains before [55,56] this could also occur for IBV/Yam. For these reasons, IBV surveillance studies should be strengthened and plans to omit IBV/Yam from the influenza vaccine panel in the near future must be considered carefully to prevent its re-emergence.

## Supplementary Material

Supplemental Material

Supplemental Material

Supplemental Material

Supplemental Material

Supplemental Material

Publication License Jul142024.pdf

Publication License Jul192024.pdf

Supplementary table 6.xlsx

Supplementary table 2.docx

Supplementary Table 4.docx

Supplementary table 5.docx

Supplementary table 3 gisaid acknowledge tables.xls

Supplementary_material-clean.docx

Supplementary table 1.docx

## Data Availability

RNAseq data generated in this study have been deposited to GEO with the accession number GSE197143.
