# Supplementary material for: Molecular characterisation of influenza B virus from the 2017/18 season in primary models of the human lung reveals improved adaptation to the lower respiratory tract
Source: Emerg Microbes Infect. 2024 Sep 9;13(1):2402868. doi: 10.1080/22221751.2024.2402868 (PMC11421153; doi:10.1080/22221751.2024.2402868)
Supplement: Supplemental Material [file TEMI_A_2402868_SM2761.pdf]

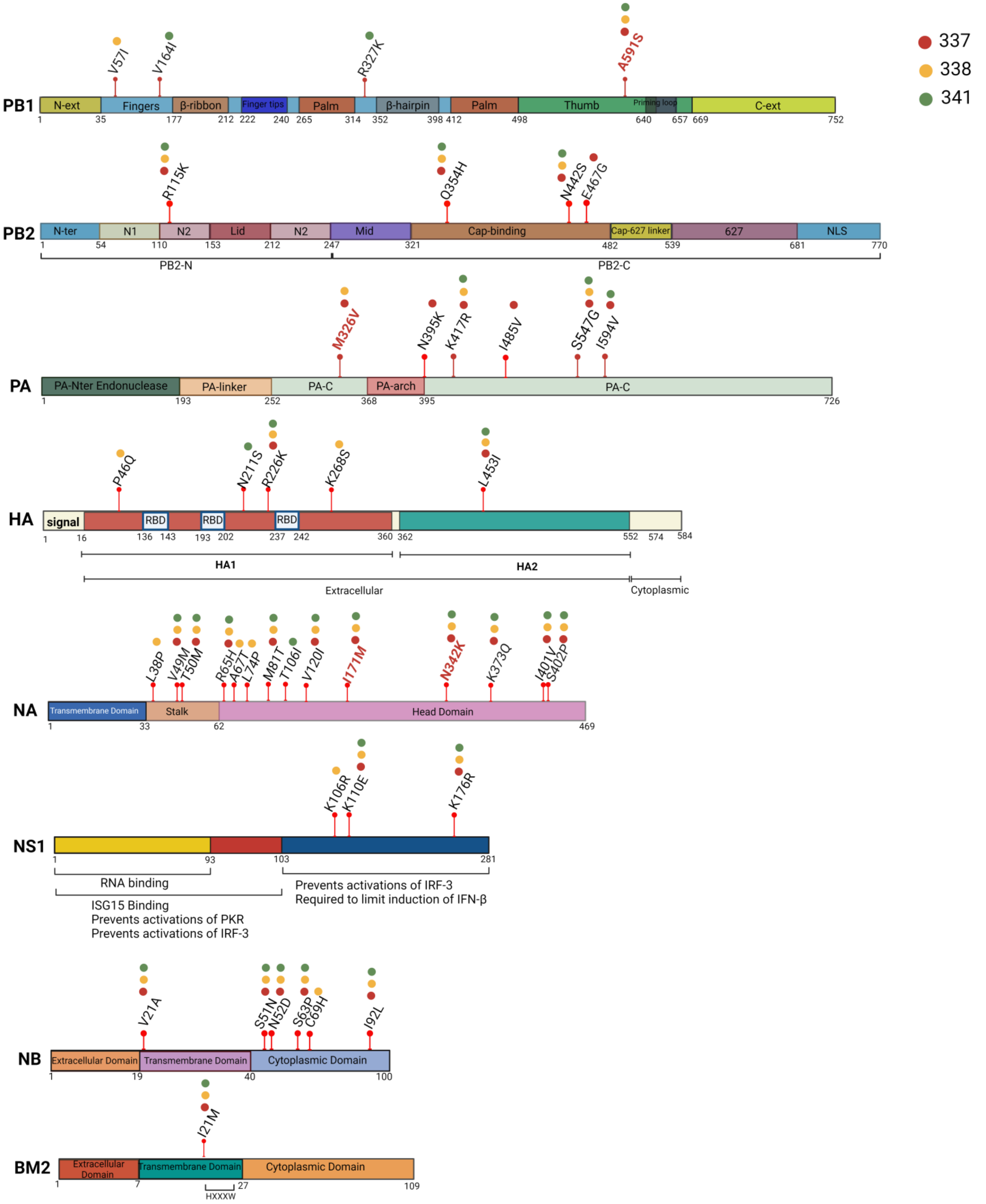

Supplementary Figure 1. Schematic diagram of the proteins of IBV and mutations in proteins of the B/18 isolates from this study compared to the B/16 isolate (black). Numbers in red refer to characteristic mutations of the 2017-2018 Clade 3A. The red dots shows mutations of B/18/337, the yellow dots shows mutations of B/18/338 and the green dots shows mutations of B/18/341
