# Supplementary material for: Molecular characterisation of influenza B virus from the 2017/18 season in primary models of the human lung reveals improved adaptation to the lower respiratory tract
Source: Emerg Microbes Infect. 2024 Sep 9;13(1):2402868. doi: 10.1080/22221751.2024.2402868 (PMC11421153; doi:10.1080/22221751.2024.2402868)
Supplement: Supplemental Material [file TEMI_A_2402868_SM2758.pdf]

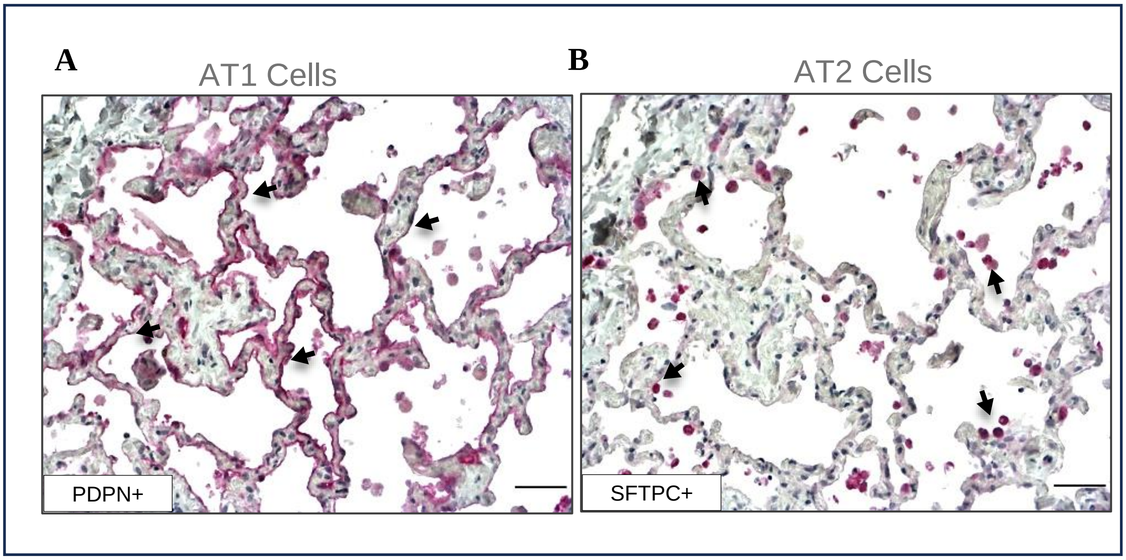

Supplementary Figure 2. Immunohistochemical staining of Podoplanin (PDPN) as a marker for AT1 cells and Prosurfactant protein C (SFTPC) as a marker of AT2 cells in un-infected explants at 37°C, 48hpi (n≥3). AT1 cells line the alveolar wall and have an elongated morphology (pink) (A) that allows gas exchange. AT2 cells are progenitor cells with cuboidal morphology (pink)(B) that can transform into AT1 cells when needed.
