# Supplementary material for: Molecular characterisation of influenza B virus from the 2017/18 season in primary models of the human lung reveals improved adaptation to the lower respiratory tract
Source: Emerg Microbes Infect. 2024 Sep 9;13(1):2402868. doi: 10.1080/22221751.2024.2402868 (PMC11421153; doi:10.1080/22221751.2024.2402868)
Supplement: Supplemental Material [file TEMI_A_2402868_SM2757.pdf]

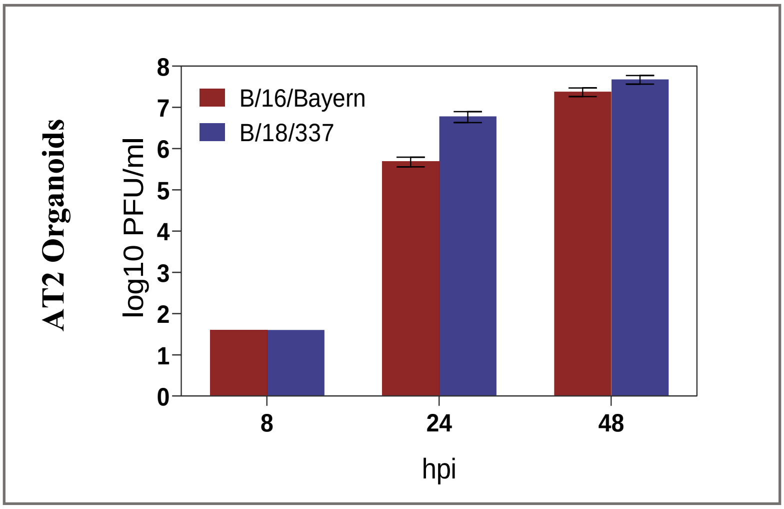

Supplementary Figure 3. Replication of B/16 and B/18/337 in lung organoids. (A) AT2-derived lung organoids were infected with MOI 0,01 at 33°C (n≥2) showing almost same replication kinetics of the B\16 and B\18 isolates.
