# Supplementary material for: Molecular characterisation of influenza B virus from the 2017/18 season in primary models of the human lung reveals improved adaptation to the lower respiratory tract
Source: Emerg Microbes Infect. 2024 Sep 9;13(1):2402868. doi: 10.1080/22221751.2024.2402868 (PMC11421153; doi:10.1080/22221751.2024.2402868)
Supplement: Supplemental Material [file TEMI_A_2402868_SM2756.pdf]

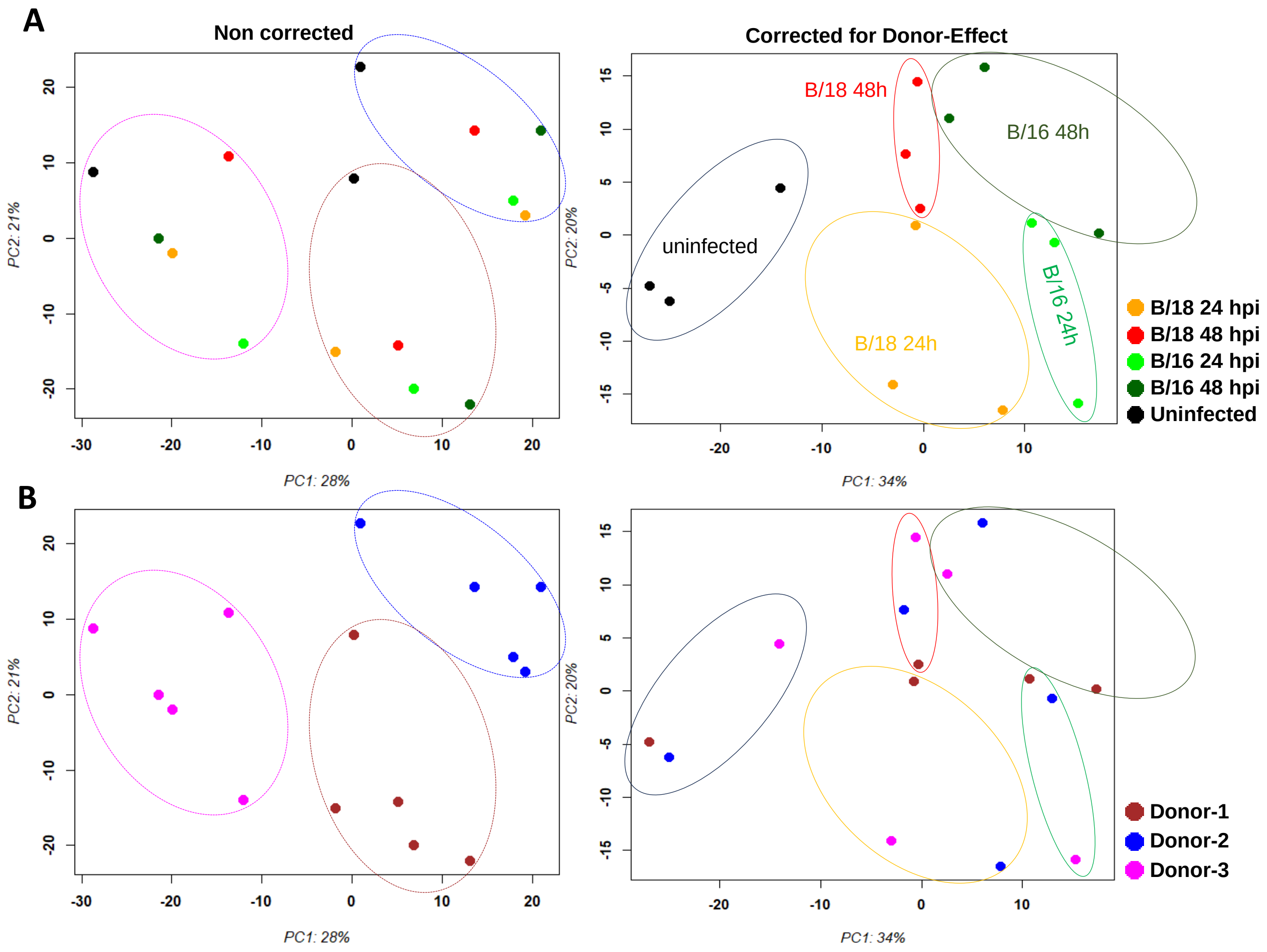

Supplementary Figure 4. Principle component analysis of 15 samples submitted to RNA sequencing. (a) PCA plots depicting donor effect before correction, with clustering of samples by treatment (top) or donors (bottom). (b) PCA plots of donor corrected data. Datasets were corrected using conventional donor-correction functions within the DESeq2/limma R-packages.
