# Supplementary material for: Molecular characterisation of influenza B virus from the 2017/18 season in primary models of the human lung reveals improved adaptation to the lower respiratory tract
Source: Emerg Microbes Infect. 2024 Sep 9;13(1):2402868. doi: 10.1080/22221751.2024.2402868 (PMC11421153; doi:10.1080/22221751.2024.2402868)
Supplement: Supplemental Material [file TEMI_A_2402868_SM2755.pdf]

A

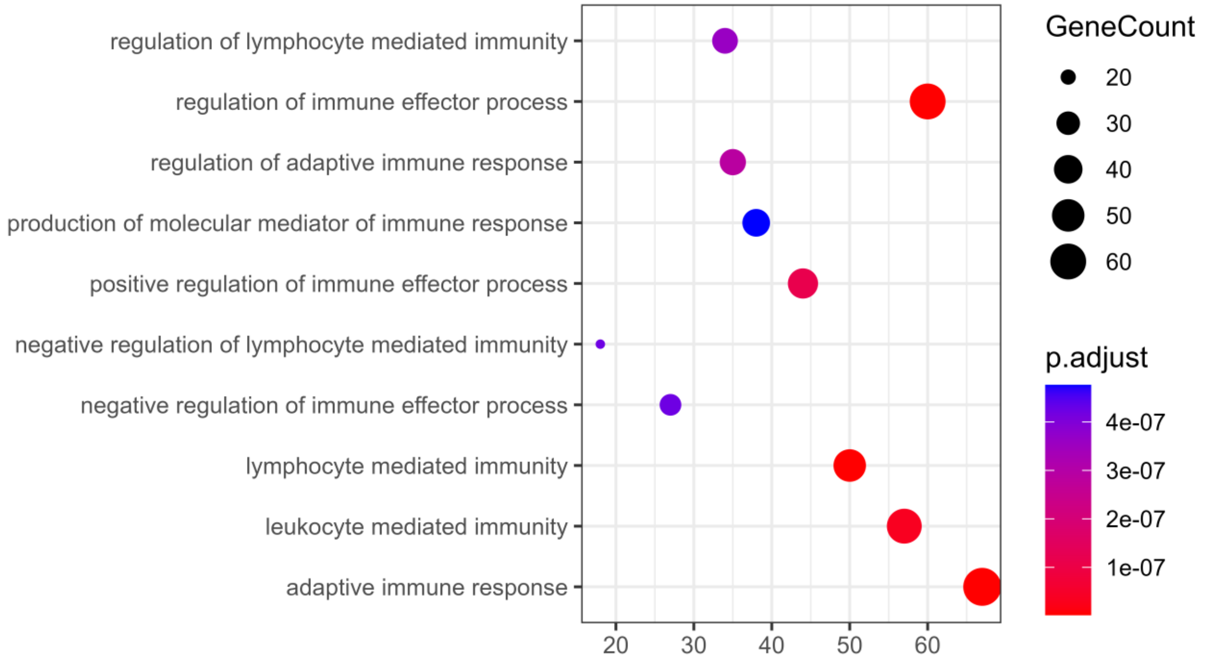

B

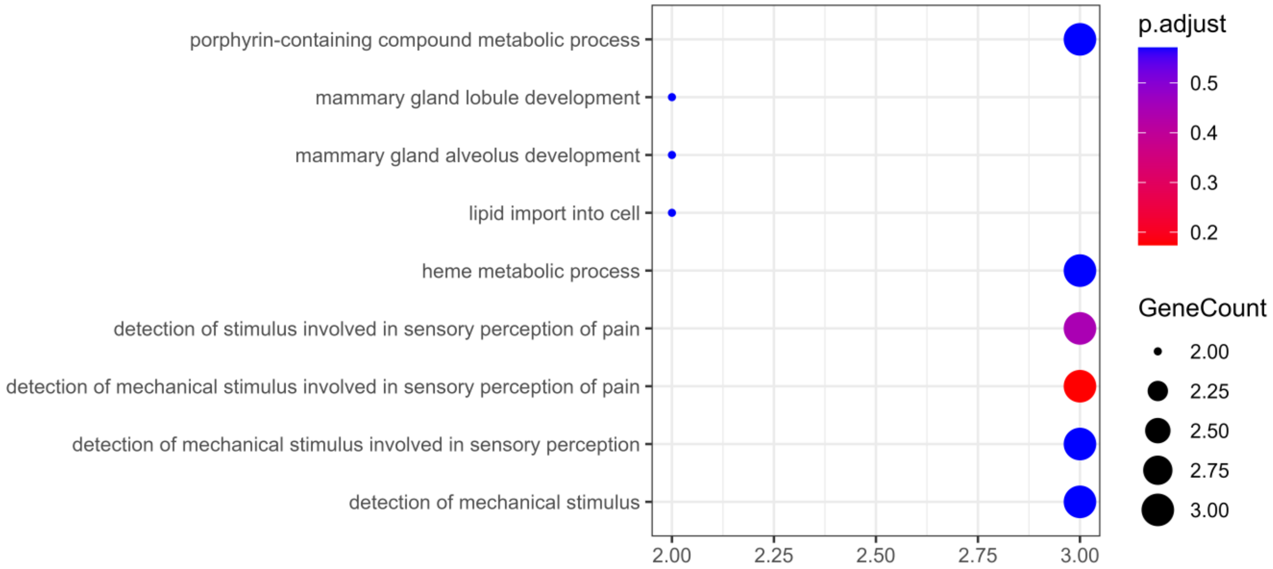

Supplementary Figure 5. Gene Ontology (GO) analysis of DEGs expressed by B/16 (A) and B/18/338 (B) generated using ClusterProfiler function over Biological Processes (BP) module; Top 10 biological processes were visualized in terms of gene counts and adjusted P.values using ggplot2 function. The 554 and 221 DEGs expressed by B/16 isolate at 24 hpi and 48 hpi respectively (as depicted in Fig. 5E) were together regarded as DEGs differentially expressed by B/16 for this GO analysis. A similar approach was adopted for the 102 and 9 DEGs expressed by B/18/338 isolate at 24 hpi and 48 hpi respectively.
