# Supplementary material for: Molecular characterisation of influenza B virus from the 2017/18 season in primary models of the human lung reveals improved adaptation to the lower respiratory tract
Source: Emerg Microbes Infect. 2024 Sep 9;13(1):2402868. doi: 10.1080/22221751.2024.2402868 (PMC11421153; doi:10.1080/22221751.2024.2402868)
Supplement: Supplementary table 2.docx [file TEMI_A_2402868_SM3740.docx]

Table 2. Meta data of patients from whom viruses were isolated

| Isolate | Age (year) | Clinical Definition | Hospitalization | Risk Group | Symptoms |
| --- | --- | --- | --- | --- | --- |
| B/Münster/2018/337 | 23 | ILI* | No | No | Asthma,thoracic pain,dyspnea, headache,fever |
| B/Münster/2018/338 | 4 | ILI* | No | No | URT infection,transient hypoglycemia |
| B/Münster/2018/341 | 84 | ILI* | No | Yes | Concentration disorders,fever, dry irritating cough |

* influenza-like illnesses
