# Supplementary material for: Molecular characterisation of influenza B virus from the 2017/18 season in primary models of the human lung reveals improved adaptation to the lower respiratory tract
Source: Emerg Microbes Infect. 2024 Sep 9;13(1):2402868. doi: 10.1080/22221751.2024.2402868 (PMC11421153; doi:10.1080/22221751.2024.2402868)
Supplement: Supplementary Table 4.docx [file TEMI_A_2402868_SM3739.docx]

**Table 4.** Clinical data of lung tissue donors

| **Donor** | **Age** | **Gender** | **Lung Region** | **Diagnose** |
| --- | --- | --- | --- | --- |
| 1 | 69 | Female | Right-Upper Lobe | Lung pleomorphic carsinoma |
| 2 | 71 | Male | Right-Upper Lobe | Non-small cell adenocarsinoma |
| 3 | 60 | Male | Right-Lower Lobe | Non-small cell adenocarsinoma |
| 4 | 67 | Female | Left-Lower Lobe | Lung Tumor |
| 5 | 67 | Female | Left Lobe | Lung Carsinoma |
| 6 | 78 | Female | Left-lower Lobe | Lung Carsinoma |
| 7 | 37 | Male | Right-Lower Lobe | Metastasis in the right lower lobe of the lung |
| 8 | 57 | Male | Right-Upper Lobe | No information |
| 9 | 77 | Male | Left-Upper Lobe | Pulmonary emphysema |
