# Supplementary material for: Molecular characterisation of influenza B virus from the 2017/18 season in primary models of the human lung reveals improved adaptation to the lower respiratory tract
Source: Emerg Microbes Infect. 2024 Sep 9;13(1):2402868. doi: 10.1080/22221751.2024.2402868 (PMC11421153; doi:10.1080/22221751.2024.2402868)
Supplement: Supplementary table 5.docx [file TEMI_A_2402868_SM3738.docx]

| Donor | Sample ID | Tissue ID | Infection Status | Virus | Hpi |
| --- | --- | --- | --- | --- | --- |
| 1 | FluB_337_24hpi | Hulu 285 | Infected | B\18\337 | 24 |
|  | FluB_337_48hpi | Hulu 285 | Infected | B\18\337 | 48 |
|  | FluB_Bay_24hpi | Hulu 285 | Infected | B\16 | 24 |
|  | FluB_Bay_48hpi | Hulu 285 | Infected | B\16 | 48 |
|  | Mock_48hpi | Hulu 285 | Uninfected |  | 48 |
| 2 | FluB_337_24hpi | Hulu 288 | Infected | B\18\337 | 24 |
|  | FluB_337_48hpi | Hulu 288 | Infected | B\18\337 | 48 |
|  | FluB_Bay_24hpi | Hulu 288 | Infected | B\16 | 24 |
|  | FluB_Bay_48hpi | Hulu 288 | Infected | B\16 | 48 |
|  | Mock_48hpi | Hulu 288 | Uninfected |  | 48 |
| 3 | FluB_337_24hpi | Hulu 294 | Infected | B\18\337 | 24 |
|  | FluB_337_48hpi | Hulu 294 | Infected | B\18\337 | 48 |
|  | FluB_Bay_24hpi | Hulu 294 | Infected | B\16 | 24 |
|  | FluB_Bay_48hpi | Hulu 294 | Infected | B\16 | 48 |
|  | Mock_48hpi | Hulu 294 | Uninfected |  | 48 |

**Table 5.** Sample details used in RNAseq
