# Supplementary material for: Molecular characterisation of influenza B virus from the 2017/18 season in primary models of the human lung reveals improved adaptation to the lower respiratory tract
Source: Emerg Microbes Infect. 2024 Sep 9;13(1):2402868. doi: 10.1080/22221751.2024.2402868 (PMC11421153; doi:10.1080/22221751.2024.2402868)
Supplement: Supplementary_material-clean.docx [file TEMI_A_2402868_SM3733.docx]

## ***Generation of AT2 derived organoids from human lung tissues***

AT2-derived lung organoids were generated based on published protocol by using human lung tissues [17]. Briefly, tissues were digested (500 U/ml Collagenase I (Thermo Fisher Scientific, Waltham, USA), 5 U/ml Dispase II (Thermo Fisher Scientific, Waltham, USA), and 1 U/ml DNase (AppliChem, Darmstadt, Germany) in Hanks’ Balanced Salt Solution (HBSS, Thermo Fisher Scientific, Waltham, USA) supplemented with 10 µM Y-27632 dihydrochloride (Tocris Bioscience, Avonmouth, UK)). After red blood cell lysis (Miltenyi Biotec, Bergisch Gladbach, Germany) and filtering steps, cells were resuspended with staining solution (Advanced DMEM/F12 (Thermo Fisher Scientific, Waltham, USA) with 1 mM HEPES (Thermo Fisher Scientific, Waltham, USA), 1% (v/v) GlutaMAX™ (Thermo Fisher Scientific, Waltham, USA), 5% (v/v) FCS (Capricorn, Ebsdorfergrund, Germany), N-2 Supplement (1X), B-27® Supplement (1X)( Thermo Fisher Scientific, Waltham, USA), 5 mM Nicotinamide (Sigma-Aldrich, St. Louis, USA), 1.25 mM N-Acetylcysteine (Sigma-Aldrich, St. Louis, USA) and 10 µM Y-27632 dihydrochloride). For isolation of HTII-280^+^ cells, cell suspension was stained with PE/Dazzle594-conjugated mouse anti-human epithelial cell adhesion molecule (EpCAM, CD326**)** (324232, Biolegend, USA) IgG antibody (1:200 diluted in staining solution) and mouse anti-HT2-280 IgM antibody ((Terrace Biotech, TB-27AHT2-280), 1:50 diluted in staining solution) for 30 min on ice. After washing with ADF++ (Advanced DMEM/F-12 supplemented with 1 mM HEPES, 1% (v/v) GlutaMAX™ 5% FCS) cells were resuspended in 1 ml staining buffer containing the secondary Alexa Fluor 488 goat anti-mouse IgM antibody (1:2000 diluted in staining solution, Thermo Fisher Scientific, USA) and incubated for 30 min on ice. In parallel, control staining was performed with cells stained only with mouse anti-human CD326 IgG antibody, mouse anti-HT2-280 IgM antibody and secondary Alexa Fluor 488 goat anti-mouse antibody without anti-HT2-280 IgM antibody. After incubation with the secondary antibodies, cells were washed and resuspended in FACS buffer (DPBS supplemented with 10 µM Y-27632 dihydrochloride and B-27®Supplement (1X)). After passing cells through a 40 µm cell strainer, cell sorting was performed using a Sony SH800S cell sorter. Sorted cells were seeded in Cultrex™ (bio-techne, Minneapolis, USA) at a concentration of ~1000 cells/µl and overlaid with the organoid media supplemented with additives (final concentrations: 10% R-spondin1 conditioned media, 25 ng/ml FGF7 (PeproTech, Cranbury, USA), 100 ng/ml FGF10 (PeproTech, Cranbury, USA ), 100 ng/ml Noggin (PeproTech, Cranbury, USA), 50nM A83-01, 0,5 µM SB-202190 (Sigma-Aldrich, St. Louis, USA), 1X B27 supplement, 1,25mM N-Acetylcystein, 5 µM Nicotinamid, 100 µg/ml Primocin (InvivoGen, Toulouse, France), 3 µM CHIR99021 (Sigma-Aldrich, St. Louis, USA), 5 µM Y-27632 dihydrochloride) for 2 to 3 weeks, changing the organoid medium every 3 days.

Organoid Media

**Table 1. Media components for organoid media.**

| **Media component** | **Stock concentration** | **Final concentration** | **Dilution** |
| --- | --- | --- | --- |
| **R-spondin1** | conditioned media | 10% |  |
| **FGF7** | 100 µg/ml | 25 ng/ml | 1:4000 |
| **FGF10** | 100 µg/ml | 100 ng/ml | 1:1000 |
| **Noggin** | 100 µg/ml | 100 ng/ml | 1:1000 |
| **A83-01** | 500 µM | 50 nM | 1:500 |
| **SB-202190** | 10 mM | 0.5 µM | 1:2000 |
| **B27 supplement** | 50X | 1X | 1:50 |
| **N-Acetylcystein** | 125 mM | 1.25 mM | 1:100 |
| **Nicotinamid** | 1 M | 5 µM | 1:200 |
| **Primocin** | 50 mg/ml | 100 µg/ml | 1:500 |
| **CHIR99021** | 3 mM | 3 µM | 1:1000 |
| **Y-27632 dihydrochloride** | 5 mM | 5 µM | 1:1000 |
| **Dissolved in ADF (supplemented with 1 mM HEPES and 1% (v/v) GlutaMAX™)** | | | |

***Preparing Suspension Culture of Organoids for Infection***

Mature human AT2-derived epithelial lung organoids were transferred to an ECM-free suspension culture one week before infection. The organoids were washed once with DPBS and released from the extracellular matrix (ECM) by adding Cultrex™ Organoid Harvesting Solution (bio-techne, Minneapolis, USA) for 30 to 90 min with moderate shaking. Cultrex™ Organoid Harvesting Solution was added at a 10X volume, Cultrex™ ECM to Cultrex™ Organoid Harvesting Solution ratio. Organoids were centrifuged and then resuspended in organoid media supplemented with 5 µM Y-27632 dihydrochloride and transferred to 24-well plates coated with Pluronic® F-127 to prevent organoids from adhering to culture plates. Every 3 to 4 days, a media change was performed.

***Infection of AT2 Derived Epithelial Lung Organoids***

After 7 days in suspension culture, organoids were infected with B/16 and B/18/337 isolates. To determine cell counts, mature organoids were dissociated into single cells using 1 ml 1X TrypLE™ Express Enzyme and incubated for 15 min at 37°C. After centrifugation at 300xg and 4°C for 5 min, organoids were resuspended in 1 ml ADF++ supplemented with 5 µM Y-27632 dihydrochloride and a single cell suspension was produced by resuspending 2 to 3 times with a disposable syringe with needle (27G). Cells were counted and the required amount of virus was calculated to infect with an MOI of 0.01

For infection, organoids were collected and centrifuged at 300xg and 4°C for 5 min. After resuspending organoids in 1 ml/well ADF++ supplemented with Y-27632 dihydrochloride (5 µM), organoids were broken up by 2 to 3 times resuspension with a disposable syringe with needle (27G) to increase the susceptibility of viruses. The organoid fragments were incubated with virus inoculum for 1 h at 33°C and 5% CO_2_. After washing once with 1 ml/well ADF++, organoids were resuspended in fresh organoid media supplemented with 5 µM Y-27632 dihydrochloride and 3 µM CHIR99021. Supernatants were collected at 8, 24 and 48 hpi and classical plaque assay was performed to determine the number of infectious virus particles.
