# Supplementary material for: Molecular characterisation of influenza B virus from the 2017/18 season in primary models of the human lung reveals improved adaptation to the lower respiratory tract
Source: Emerg Microbes Infect. 2024 Sep 9;13(1):2402868. doi: 10.1080/22221751.2024.2402868 (PMC11421153; doi:10.1080/22221751.2024.2402868)
Supplement: Supplementary table 1.docx [file TEMI_A_2402868_SM3732.docx]

Table 1. IBV-Yamagata primers used in whole genome sequencing

| **Viral Segment** | **Forward primer** | **Reverse primer** |
| --- | --- | --- |
| PB1 | AGCAGAAGCGGAGCCTTTAAG | AGTAGAAACACGAGCCTTTTTTC |
| PB2 | AGCAGAAGCGGAGCGTTTTCA | AGTAGAAACACGAGCATTTTT |
| PA | AGCAGAAGCGGTGCGTTTGAT | AGTAGAAACACGTGCATTTTT |
| HA | AGCAGAAGCAGAGCATTTTCTAATATCC | AGTAGTAACAAGAGCATTTTTCAATAACG |
| NP | AGCAGAAGCACAGCATTTTCTTG | AGTAGAAACAACAGCATTTTTTAC |
| NA | AGCAGAAGCAGAGCATCTTCTC | AGTAGTAACAAGAGCATTTTTCAGAAAC |
| M | AGCAGAAGCACGCACTTTCTT | AGTAGAAACAACGCACTTTTTC |
| NS | AGCAGAAGCAGAGGATTTGTT | AGTAGTAACAAGAGGATTTTTA |
